# Systemic deficiency of vitronectin is associated with aortic inflammation and plaque progression in ApoE‐Knockout mice

**DOI:** 10.1096/fba.2021-00108

**Published:** 2021-11-19

**Authors:** Devasmita Chakravarty, Aleepta Guha Ray, Vivek Chander, Ulaganathan Mabalirajan, Prakash Chandra Mondal, Khawer N. Siddiqui, Bishnu Prasad Sinha, Aditya Konar, Arun Bandyopadhyay

**Affiliations:** ^1^ Department of Cell Biology and Physiology CSIR‐Indian Institute of Chemical Biology Kolkata India; ^2^ Department of Cardiology Apollo Hospitals Kolkata India; ^3^ Cardiac Research Division Ruby General Hospital Kolkata India; ^4^ Department of Cancer Biology and Inflammatory Disorder CSIR‐Indian Institute of Chemical Biology Kolkata India; ^5^ Department of Laboratory Animal Facility CSIR‐Indian Institute of Chemical Biology Kolkata India

**Keywords:** atherosclerosis, cholesterol, endothelium, inflammation, plaque

## Abstract

Optimal cell spreading and interplay of vascular smooth muscle cells (VSMC), inflammatory cells, and cell adhesion molecules (CAM) are critical for progressive atherosclerosis and cardiovascular complications. The role of vitronectin (VTN), a major cell attachment glycoprotein, in the pathogenesis of atherosclerosis remains elusive. In this study, we attempt to examine the pathological role of VTN in arterial plaque progression and inflammation. We found that, relative expression analysis of VTN from the liver of Apolipoprotein E (ApoE) Knockout mice revealed that atherosclerotic progression induced by feeding mice with high cholesterol diet (HCD) causes a significant downregulation of VTN mRNA as well as protein after 60 days. Promoter assay confirmed that cholesterol modulates the expression of VTN by influencing its promoter. Mimicking VTN reduction with siRNA in HCD fed ApoE Knockout mice, accelerated athero‐inflammation with an increase in NF‐kB, ICAM‐1, and VCAM‐1 at the site of the plaque along with upregulation of inflammatory proteins like MCP‐1 and IL‐1β in the plasma. Also, matrix metalloprotease (MMP)‐9 and MMP‐12 expression were increased and collagen content was decreased in the plaque, in VTN deficient condition. This might pose a challenge to plaque integrity. Human subjects with acute coronary syndrome or having risk factors of atherosclerosis have lower levels of VTN compared to healthy controls suggesting a clinical significance of plasma VTN in the pathophysiology of coronary artery disease. We establish that, VTN plays a pivotal role in cholesterol‐driven atherosclerosis and aortic inflammation and might be a useful indicator for atherosclerotic plaque burden and stability.

**Abbreviations**
ACS: acute coronary syndrome
CAD: coronary artery disease
CAM: cell adhesion molecules
CVD: cardiovascular disease
HCD: high cholesterol diet
LDL: low‐density lipoprotein
NCD: normal chow diet
SREBP: sterol regulatory element‐binding protein
VTN: vitronectin

## 1 INTRODUCTION

Atherosclerosis is a lipid‐driven chronic inflammatory disease affecting the intimal layer of the large and medial arteries and is the most frequent cause of cardiovascular complications.^1^ Atherosclerotic research suggests a highlighted role of inflammation in the aorta, where the interplay between the pro‐and anti‐inflammatory mechanisms seems to decide the fate of the plaque.^2^, ^3^, ^4^ Therefore, understanding the factors, governing the athero‐inflammation is essential for managing patients with CVDs.

Cholesterol has been considered as the genetic and environmental promoter of atherosclerotic disease.^5^ The initiating steps in developmental atherosclerosis are caused by the accumulation of LDL‐cholesterol (LDL‐C) followed by its sequestration in the subendothelial space, where it gets oxidized.^6^ Ox‐LDL induces inflammatory responses in the aorta.^7^, ^8^ The sub‐endothelial trigger of inflammation occurs in the areas of non‐laminar blood flow while the initial stages of plaque formation are mediated through endothelial cell adhesion molecules (CAM).^9^, ^10^, ^11^ The secretion of adhesion molecules by a dysfunctional endothelium aid in the infiltration of cells to the sub‐endothelium followed by the formation of foam cells. Gradually a necrotic core is formed which if not cleared timely, atherothrombotic vascular diseases such as stroke or myocardial infarction results.^12^ Different adhesion molecules have been described and studied in the context of atherosclerosis. Most of the CAMs are present in the vascular endothelium and on the leukocytes, and their coordinated interactions are required for the successful recruitment of inflammatory cells from the blood to the site of injury, that is, the vessel in atherosclerosis. If the triggering irritants are removed, the inflammation phase is replaced with the resolution phase. On the other hand, if those irritants persist, repeated inflammation‐resolution cycles lead to tissue remodeling.^13^ However, some adhesion molecules not only orchestrate the inflammation but also enhance the tissue remodeling. Vitronectin (VTN) is one such CAM and is a multifunctional protein found predominantly in plasma and extracellular matrix.^14^ Although it is crucial in cell spreading, it also participates in wound healing, where resolution of inflammation is required. Although in general it is believed that VTN may have a pro‐inflammatory role in orchestrating the inflammation, this view seems to be incomplete. For example, in inflammatory airway diseases like asthma and COPD where tissue remodeling is a major phenomenon, VTN levels are found to be reduced.^15^ However, there are no reports regarding the functional involvement of VTN in pathological atherosclerosis in the literature.

In this study, we have provided clear evidence that systemic deficiency of VTN, is associated with inflammation and plaque progression in Apolipoprotein E (ApoE) Knockout mice. We show a reciprocal relationship of VTN with cholesterol load. Moreover, VTN downregulation drives towards unresolved inflammation and rupture‐prone plaque by accumulating cells and overexpressing adhesion molecules. The inverse correlation of VTN with the severity of coronary artery disease (CAD) is also established in human subjects. Overall, the present study demonstrates a critical association of VTN with the ongoing inflammatory process in the aorta signifying its novel role in CAD.

## 2 METHODS

### 2.1 Animal studies

Apolipoprotein E Knockout mice and Peroxisome proliferator activated receptor alpha (PPARα) Knockout mice were received from the CSIR‐Centre for Cellular and Molecular Biology and Jackson Laboratory, U.S.A, respectively. Mice colony was maintained in the institutional animal house. The protocol for the animal experimentation was approved by the Animal Ethics Care and Institutional Review Board (reference IICB/AEC/Meeting/2018/Aug and IICB/AEC/Meeting/May/2019/9) and the animals were handled in accordance with the Committee for the Purpose of Control and Supervision of Experiments on Animals (CPCSEA), Ministry of Social Justice, and Government of India (registration no. 147/1999/CPCSEA) which complies with NIH Guide for the Care and Use of Laboratory Animals. All animals used for experiments were acclimatized at least a week before starting of experiments and were age‐matched (6–8 weeks old). The mice have housed in individually ventilated cages with 12‐h light, 12‐h dark cycle and were fed the diets ad libitium.

A high cholesterol diet (HCD, cat. no. D12336) was purchased from Research Diets having 1.25% cholesterol. For time point studies, ApoE Knockout mice (*n* = 6 in each group) were fed with HCD or NCD for 15, 30, 45, and 60 days. PPARα Knockout mice (*n* = 6 in each group) were fed with HCD or normal chow diet (NCD) for 45 and 60 days. For in‐vivo experiments with atorvastatin, ApoE knockout mice (*n* = 6 in each group) were fed with HCD for 30 days, post which the diet was switched to NCD. The Control group was administered phosphate buffered saline (PBS) and the test group was administered with 50 mg/kg atorvastatin, once a day, for 4 weeks. At the end of the experimental duration, animals were anesthetized by an intraperitoneal injection of 100 mg/kg ketamine with 10 mg/kg xylazine hydrochloride and sacrificed via cervical dislocation and blood was collected by heart puncture.

In knockdown studies (*n* = 6 in each group), at the end of the protocol, mice were euthanized with an intraperitoneal injection of a pentobarbital overdose (100 mg/kg) and blood was drawn from the right ventricle, after which perfusion was done. In all the experiments, male mice were used.

### 2.2 Knockdown studies in animals

Vitronectin silencing was performed in ApoE knockout mice. For the si‐RNA mediated downregulation of VTN, gene‐specific si‐RNA was purchased from Ambion, ready to use (cat. no. 4457308) and scramble (cat. no. 445289). Invivofectamine 3.0 from Thermofischer Scientific was used for the in vivo delivery of the siRNA, which has a high transfection efficiency in the liver.^16^ ApoE knockout mice were fed with HCD for 30 days after which either 4nM of scramble or VTN‐siRNA was complexed with invivofectamine and was injected via the tail vein of the mice at an interval of 10 days.

### 2.3 Isolation of plasma

Blood was collected in ethylenediamine tetraacetic acid (EDTA)‐Vacutainers (human) or EDTA‐Microtainers (mice) from Becton Dickinson and mixed immediately after collection. It was allowed to stand for at least 2 h at 4°C for the separation of plasma, which was collected in 1.5 ml tubes and centrifuged at 350 *g* for 20 min. Supernatants were collected and stored in aliquots at −80°C.

### 2.4 Histopathological analysis and Inflammation scoring

Mice were perfused with 4% paraformaldehyde via the left cardiac ventricle and then tissues were excised out. Tissues were then dehydrated and embedded in paraffin. The paraffin‐embedded tissue sections were cut into 5‐micron size and stained with hematoxylin and eosin.^17^ The intima‐media thickness (IMT) was measured using ImageJ software by an investigator blinded to group assignment.^18^ To access the aortic inflammation, slides were numbered randomly and given to blind investigators for evaluation. The score was given from 0–4 as described previously.^19^, ^20^ Briefly, score 0 was assigned when there were no signs of inflammation either in the perivascular area or in the aortic layers, score 1 was assigned when there was perivascular infiltration of inflammatory cells, score 2 for small clusters of inflammatory cells in the tunica media, score 3 for large clusters of inflammatory cells in tunica media and also in intima and score 4 for vascular obstruction.

### 2.5 Picrosirius red staining and morphometry

Paraffin sections were deparaffinized and rehydrated followed by incubation with picrosirius red for 1 h. After 1 h, the slides were washed in two changes of 2.5% glacial acetic acid, dehydrated, dried, and mounted. The quantitative morphometry in picrosirius red‐stained aorta sections was performed using the ImageJ software version 1.8 (https://imagej.nih.gov/ij/download.html) to measure collagen content.^21^ Briefly, picrosirius red‐stained aorta sections were microphotographed and the individual image was opened via ImageJ software, split into red, blue, and green channel images. Green channel image was opened to adjust the threshold such a way that only positively stained area was given pseudocolor and using automated particle analysis the positively stained area was measured and expressed as % positive stained area.

### 2.6 Immunofluorescence

Mice were sacrificed after perfusion and tissues were dehydrated and embedded in paraffin. Microtomy was then performed and tissues were stained with antibodies as previously described.^22^ Briefly, tissue sections were processed for staining with the following antibodies against p65‐NF‐KB (1:50) (cat. no. sc‐33020; Santa Cruz), ICAM‐1 (1:50) (cat. no. sc‐7891; Santa Cruz), and VCAM‐1 (1:100) (cat. no. Ab134047; Abcam), α‐smooth muscle actin (α‐SMA) (1:25) (cat. no. MAB1420; R & D Systems), matrix metalloprotease (MMP)‐12 (1:50) (cat. no. sc‐30072; Santa Creuz), MMP‐9 (1:50) (cat. no. sc‐393859; Santa Crueuz). Following incubation in primary antibody overnight, sections were incubated with Alexa Fluor conjugated secondary antibodies (1:300) (cat. no. A21206, cat. no. A10037; Invitrogen). Counterstaining with DAPI was performed. Images were captured in TCSSP8 Lightning Confocal Microscope, Leica Microsystems.

### 2.7 MTT assay

HepG2 cells were seeded in 96‐well plate. It was serum starved for 6 h followed by treatment with different cholesterol concentration 25, 50, 75, and 100 µM for 48 h with replenishment after 24 h. After 48 h 3‐(4,5‐dimethylthiazol‐2‐yl)‐2,5‐diphenyltetrazolium bromide (MTT) reagent was added into the wells and incubated for 3 h at 37°C. Absorbance was then taken at 590 nm. Relative cell survival was then calculated.

### 2.8 Western blotting

Cells were lysed and total protein was estimated by using DC™ Protein Assay Kit (Bio‐Rad). Mice plasma was diluted with PBS in a ratio of 1:10 before gel separation. Proteins were resolved in 10% sodium dodecyl sulphate–polyacrylamide gel electrophoresis and transferred onto polyvinylidene fluoride membrane (Millipore). Membranes were incubated in 5% nonfat dry milk, followed by incubation with a primary and secondary antibody with three alternate wash. Blots were finally incubated with NBT and BCIP in alkaline phosphatase buffer until the bands were visible. The primary antibodies used for the study were anti‐vitronectin (1:1,000) (cat no. 45139; Abcam), anti‐transferrin (1:10,000) (cat no. Ab82411; Abcam), anti‐β‐actin (1:10,000) (cat. no. A5441; Sigma), anti‐ApoA2 (1:1000) (cat. no. A14690; Ab clonal), anti‐ApoA1 (1:1000) (cat no. sc‐376818; Santa Cruz), IL‐1β (1:1000) (cat no. sc‐52012; Santa Cruz), MCP‐1 (1:2000) (cat no. A7277; Ab clonal). Quantitative analysis was done by Image J software. The ratio of levels of experimental protein/loading control was used for the evaluation of relative levels of the protein.

### 2.9 Enzyme Linked Immunosorbent Assay

Estimation of VTN in healthy as well as ACS subjects was conducted by commercially available colorimetric assay kit (Takara Human VTN EIA kit, cat. no. MK103) by diluting human plasma at 1:1500, by sandwich Enzyme Linked Immunosorbent Assay (ELISA) method as per the manufacturer's instructions. The detection limit of the kit was 5 ng/ml for VTN. The intra‐assay CV was <10% and the inter‐assay CV was <5% in all the ELISA. The estimation of mice VTN level was determined by ELISA kit from Molecular Innovations (cat no. MVNKT‐TOT). The minimum detectable dose was 0.013 ng/ml.

### 2.10 Quantitative PCR

Total RNA from cell or tissue homogenates was isolated using Trizol (Invitrogen) and was quantified by using Luna® Universal One‐Step RT qPCR kit (NEB) (cat no. E3005L). The reaction was set up in AB Real‐Time 7500 Fast system. RT‐PCR primers were designed using Primer Bank. The primer sequences are mouse GAPDH F(5′‐TGGCCTTCCGTGTTCCTAC‐3′), R(5′‐GAGTTGCTGTTGAAGTCGCA‐3′), mouse VTN(5′‐C CACAGACGCAGCCAGAG‐3′), R(5′‐GCAGGTACAAGCC AGTCCAT‐3′), human GAPDH F(5′‐GGGAAGCTTGTC AT CAATG GA‐3′), R(5′‐TCTCGCTCCTGGAAGATGGT‐3′), human VTN F(5′‐GAGTG CAAGCCCCAAGTGAC‐3′), R(5′‐GCCATCGTCATAGACCGTGT‐3′). The fold change of the experimental gene was calculated and was normalized with the control gene.

### 2.11 Cell culture

HepG2 cells and human embryonic kidney 293 (HEK‐293T) cells were obtained from National Centre for Cell Sciences, Pune. HepG2 cells were maintained in MEM medium and HEK‐293T cells were maintained in DMEM media. In all experiments, cells were serum‐starved for 12 h before treatment. HepG2 cells were treated with Cholesterol (cat. no. 97900; Sisco Research Laboratories Pvt. Ltd.), Atorvastatin (cat. no. PZ0001; Sigma), and LDL was isolated from human plasma.

### 2.12 Luciferase reporter assay

The DNA fragment containing the promoter region (up to −1000 bp) of the VTN gene was cloned downstream of the luciferase gene within the pGL3‐luciferase reporter vector using primers F (5′‐GGGGTACC TTCCATTCCACAGCTGACTCC‐3′) and R(5′‐GACCTC GAGCCCATGAGGAAGAAA TTGGC‐3′). Luciferase reporter plasmids along with the promoter were co‐transfected into HEK‐293T cells, which is commonly preferred due to high transfection efficiency, using Lipofectamine 2000 (Invitrogen). One day after transfection, the media was changed to serum‐free media for 12 h, followed by 48 h of treatment with increasing concentrations of cholesterol, with replenishment at 24 h. The cells were then harvested to measure the luciferase activity using the Dual‐Luciferase Reporter Assay System (Promega).

### 2.13 Human studies

The entire protocol involving human participants was approved by the Ethics Committees of CSIR‐Indian Institute of Chemical Biology and Apollo Gleneagles Hospital, Kolkata (reference no. IEC Ref: IEC/2013/10/32). Informed consent was also obtained from each individual before blood collection. To find out the clinical correlation of VTN in human plasma we have selected a reasonable number enough for statistical analysis. There were three groups: Group A (*n* = 10) comprised of healthy control individuals who had no risk factors, normal systolic/diastolic blood pressure, normal blood and lipid profiles, normal‐electrocardiograph and echocardiograph reading with normal carotid IMT (<0.8 mm); Group B (*n* = 46) comprised of individuals with risk factors like diabetes mellitus, dyslipidemia, hypertension, CAD and smoking; and Group C (*n* = 23) comprised of the individuals having clinically diagnosed acute coronary syndrome (ACS). Subjects having other ailments such as renal failure, hepatic failure, cancer, thyroid, platelet‐related disorders, or other vascular diseases were excluded from the study. Blood samples were collected from Group C in Apollo Gleneagles Hospital after clinical confirmation of diagnosis, supplemented by electrocardiogram, Troponin T, and echocardiography. Whereas blood from age‐matched healthy subjects was collected from the local population after through routine check‐ups. Blood plasma was separated by centrifugation at 2500× g for 10 min and stored in a −80°C freezer until use.

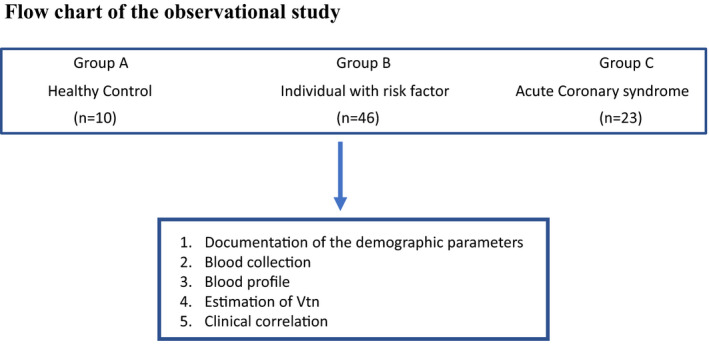

#### 2.13.1 Statistical analysis

Data are expressed as mean ± standard error of the mean (SE). Student's unpaired *t*‐test or One‐way ANOVA with *Bonferroni* post‐hoc test was performed for statistical analysis using GraphPad PRISM 8. Differences were considered statistically significant if the p‐value is less than 0.05. *p < 0.05; **p < 0.01, ***p < 0.001, ****p < 0.0001.

## 3 RESULTS

### 3.1 VTN is downregulated with progressive atherosclerosis upon feeding with HCD

**FIGURE 1:**
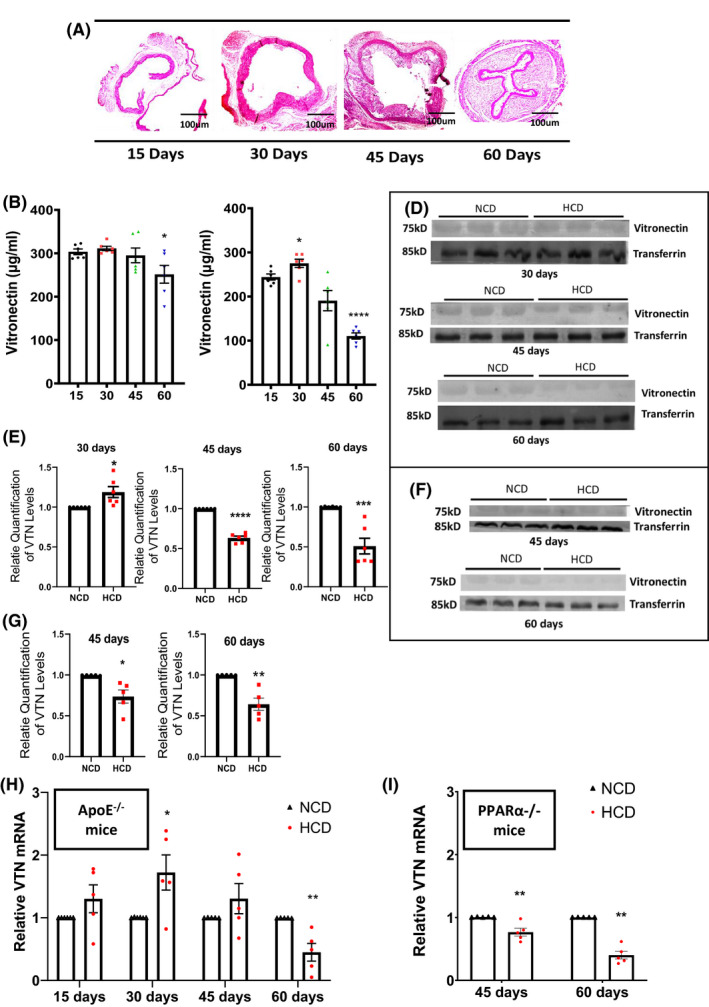
Dietary cholesterol downregulates the expression of vitronectin (VTN) (A) Representative images showing aortic lesion in ApoE^−/−^ mice fed with HCD for indicated duration (15–60 days). Quantitative estimation of plasma levels of VTN by ELISA in ApoE^−/−^ mice fed with either normal chow diet (NCD) (B) or high cholesterol diet (HCD) (C) for different duration (B *n* = 6, C *n* = 6). Western blot of VTN from plasma in ApoE^−/−^ mice (D) and PPARα^−/−^ mice (F) and its quantification graph showing relative levels of VTN (E *n* = 6, G *n* = 5). Expression of VTN in liver, normalized to GAPDH in Apo^−/−^ mice (H *n* = 5) and PPARα^−/−^ mice (I *n* = 5), unpaired *t*‐test, *p < 0.05, **p < 0.01, ***p < 0.001, ****p < 0.0001

Earlier studies have found an increase in VTN in coronary artery disease patients.^23^, ^24^ As cross‐sectional studies cannot capture the real association with the disease, we have performed time kinetic studies to check the levels of VTN with the progression of atherosclerosis. To explore any potential association between VTN and atherosclerotic plaque development, the plasma level of VTN was investigated in ApoE Knockout mice fed either with a HCD or NCD for different durations of time (15–60 days). The fatty steak deposition was apparent after 45 days of HCD feeding (Figure 1A). While no apparent changes in plasma VTN concentration were noticed in NCD‐fed ApoE knockout mice (Figure 1B), an increase in the level of VTN concentration after 30 days followed by a significant decline after 60 days (*p* < 0.0001) was observed in HCD fed mice (Figure 1C). Validation by Western blot revealed a similar pattern of VTN upregulation, followed by downregulation after HCD feeding (Figure 1D,E). To further confirm the influence of the cholesterol in regulating VTN level in vivo, the plasma levels of VTN in dyslipidemic PPARα Knockout mice were measured. As fatty streaks were apparent only after 45 days in ApoE Knockout mice, we fed PPARα Knockout mice with NCD and HCD for 45 and 60 days only. Immunoblotting analysis revealed a reduction of VTN in mice fed with HCD compared to NCD at 45 days as well as 60 days (Figure 1F,G). Since the liver is the main site of synthesis of VTN, we performed VTN mRNA expression analysis from liver of both ApoE and PPARα Knockout mice, to understand the cause of VTN downregulation in plasma. The results demonstrated the downregulation of VTN mRNA in both HCD fed mice groups (Figure 1H,I). These results establish an inverse relationship of VTN with cholesterol load in vivo.

### 3.2 Cholesterol regulates the expression of the VTN gene

**FIGURE 2:**
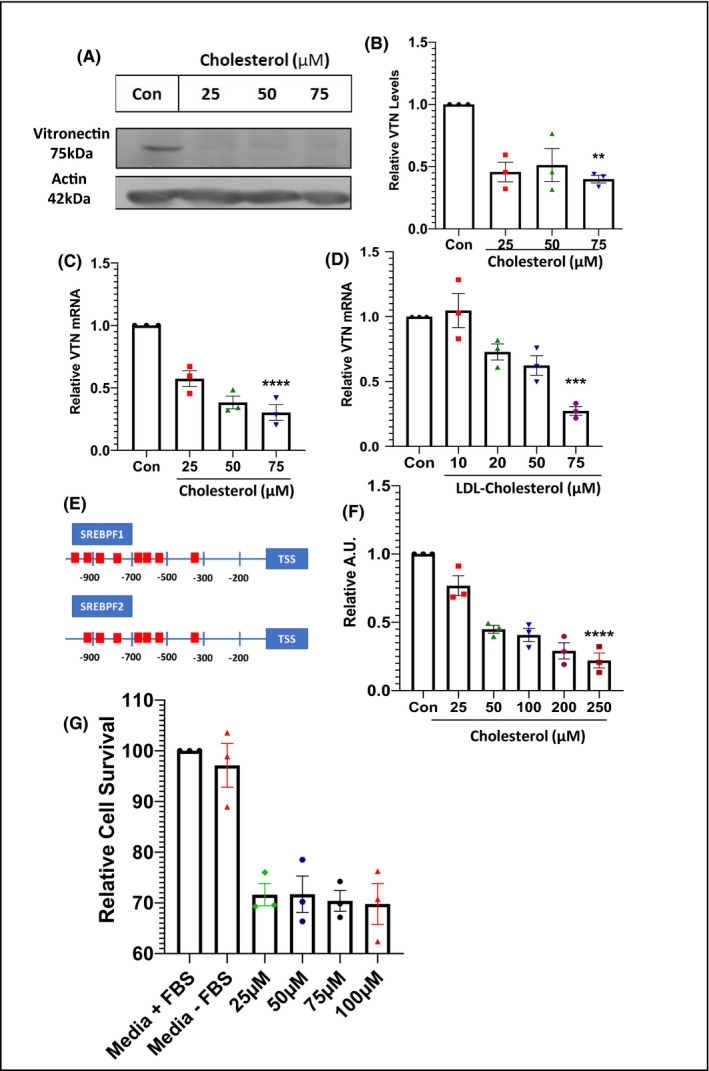
Cholesterol regulates vitronectin expression (A) Western blot of vitronectin (VTN) from HepG2 cells treated with increasing concentration of cholesterol (25, 50, 75 µM) for 48 h with replenishment at 24 h, and its relative quantification (B, *n* = 3). (C) Expression levels of VTN under similar conditions. (D) Expression level of VTN in HepG2 cells in presence of increasing concentration of LDL‐C for 48 h (*n* = 3). (E) Schematic representation of binding sites for SREBPF1 and SREBPF2 in the promoter region of human VTN gene. (F) Luciferase assay showing human VTN promoter activity in response to increasing concentration of cholesterol for 48 h in HEK293T cells (*n* = 3), one‐way ANOVA, with Bonferroni post hoc test. (G) MTT‐assay of HepG2 cells showing relative cell survival of cells at different concentrations of cholesterol, *n* = 3 *p < 0.05, **p < 0.01, ***p < 0.001, ****p < 0.0001

It is well known that free cholesterol released from the atheromatous plaque, can activate membrane attack complex (MAC) which are plaque destabilizing. On the other hand, several soluble molecules including VTN inhibit the formation of MAC‐complex and thus stabilize the atherosclerotic plaque.^25^, ^26^ So, we hypothesized that cholesterol may be directly involved in the regulation of VTN gene expression. Since VTN gene expression is reduced in mice fed with high cholesterol, the expression of VTN with increasing concentration of cholesterol was investigated in vitro in HepG2 cells. Western blot (Figure 2A,B) analysis showed a decrease in protein level with increasing cholesterol load for 48 h after a replenishment at 24 h. This was further validated by RT‐PCR analysis (Figure 2C) which also revealed a decrease in VTN mRNA level under similar conditions. To clarify whether LDL‐C is equally able to suppress VTN, HepG2 cells were treated with different doses of LDL, isolated from human plasma, and VTN gene expression was checked (Figure 2D). The results showed the same pattern of VTN downregulation. To understand the possible mechanism behind cholesterol mediated regulation of VTN expression, promoter analysis of human VTN gene sequence, using EPD (Eukaryotic Promoter Database) was performed which revealed eight SREBPF1 (−332, −593, −622, −691, −786, −841, −971 and −984) and seven SREBPF2 (−333, −592, −621, −690, −777, −841, −970) binding regions upstream of the Transcription Start Site (using Transcription Factor Motifs‐JASPER); (*p* < 0.001). A schematic diagram of the same is shown (Figure 2E). Next, to further validate the promoter‐specific interaction of cholesterol with the VTN gene, a luciferase promoter assay was performed. The results showed a dose‐dependent downregulation of luciferase activity with increasing cholesterol concentration (Figure 2F). We also performed MTT‐assay for the validation of cell viability and to demonstrate that the reduction in VTN expression is primarily dependent on cholesterol treatment, not due to cell death. Results of MTT assay shows that the survival rates of the HepG2 Cells are indifferent in different dosage of cholesterol treatment (Figure 2G). This further proves VTN downregulation is cholesterol‐dependent. Taken together these results indicate that high cholesterol load downregulates the activity of the VTN promoter.

### 3.3 Statins mediate induction of VTN expression

**FIGURE 3:**
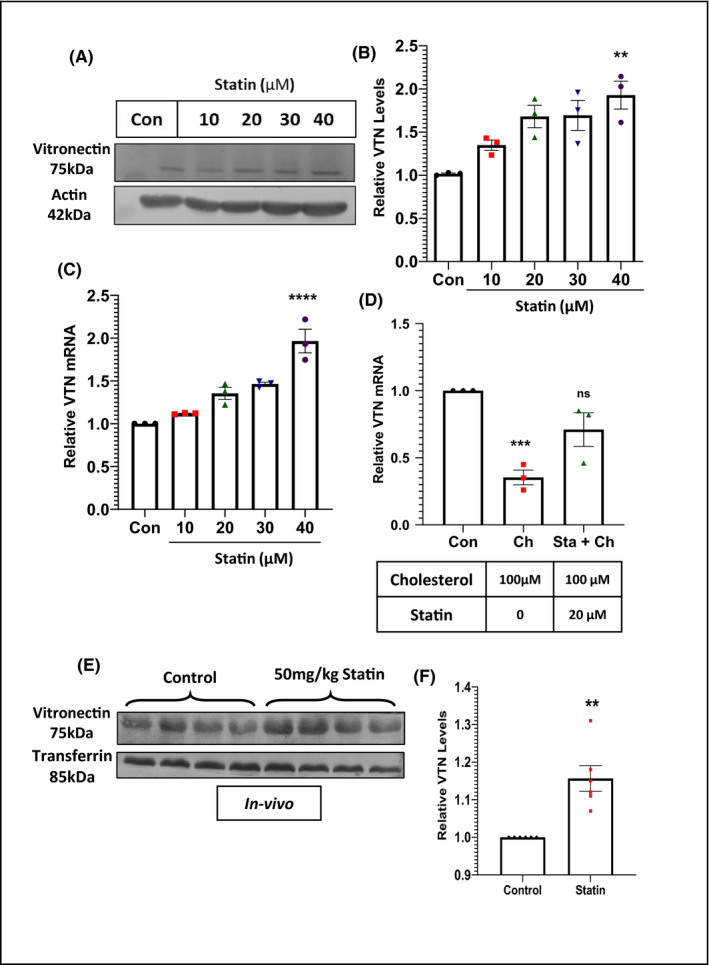
Statin upregulates expression of vitronectin (VTN) (A) Representative western blot of VTN from HepG2 cells treated with increasing concentration of Atorvastatin (10, 20, 30, 40 µM) for 48 h and its quantification (B, *n* = 3). (C) Expression level of VTN in HepG2 cells in presence of increasing concentration of atorvastatin for 48 h (*n* = 3). (D) Expression level of VTN in HepG2 cells, showing the rescue of VTN downregulation in presence of 100 µM cholesterol, with simultaneous treatment of 20 µM atorvastatin (*n* = 3). (E) Western blot showing the plasma levels of VTN in ApoE^−/−^ mice, after treatment with 50 mg/kg atorvastatin for 4 weeks (*n* = 4), one‐way ANOVA, with Bonferroni post hoc test or unpaired *t*‐test. **p < 0.05, **p < 0.01, ***p < 0.001, ns‐non significant

Statins are common medications having overwhelming evidence to lower cholesterol levels and cardiovascular complications. The most widespread among statins is atorvastatin. To further explore whether the mechanism of cholesterol‐mediated downregulation of VTN can be reversed by diminishing cholesterol levels, experiments were performed both in vitro and in vivo. HepG2 cells were serum‐starved for 12 h and were treated with increasing atorvastatin concentration for 48 h with replenishment at 24 h. Western blot (Figure 3A,B), as well as RT‐PCR analysis (Figure 3C), showed a dose‐dependent increase in VTN expression along with the increasing concentration of atorvastatin. Next, HepG2 cells were treated with 100 µM cholesterol alone or in combination with 20 µM statin for 48 h. Compared to control, a significant downregulation of VTN expression was observed when treated with 100 µM cholesterol, while there was no significant difference when 20 µM statin was added with cholesterol (Figure 3D). Furthermore, in‐vivo experiments were performed to analyze the effect of statin on VTN expression. ApoE Knockout mice were fed with HCD for 30 days, post which the diet was switched to NCD because, with the continuous supply of a 1.25% cholesterol diet, it would have been difficult for statins to alter the cholesterol levels. Following this, the treatment group was treated with 50mg/kg atorvastatin by oral gavaging, while the control mice were treated with saline, once a day, for 4 weeks. Western blot analysis from mice plasma (Figure 3E, F) showed an upregulation of VTN expression when treated with 50 mg/kg atorvastatin. Collectively, these results demonstrate that statins aid in the upregulation of VTN gene expression possibly by preserving cholesterol concentration.

### 3.4 VTN depletion promotes inflammatory cell migration and atherosclerosis in ApoE‐deficient mice

**FIGURE 4:**
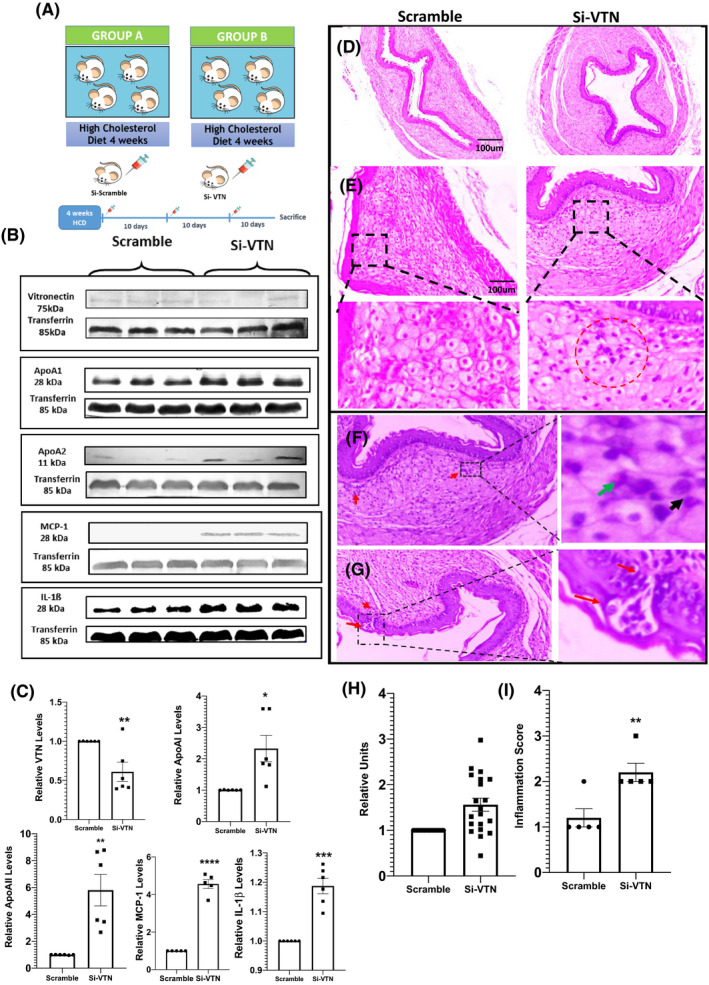
Increase in plaque size and migration of inflammatory cells in atherosclerotic plaque in vitronectin (VTN)‐deficient ApoE^−/−^ mice (A) Schematic diagram showing the experimental design of VTN silencing by si‐RNA. (B) Western blot showing plasma level of VTN, ApoA‐I, ApoA‐II, MCP‐1 and IL‐1β in scramble and VTN siRNA treated ApoE^−/−^ mice (*n* = 6) and their respective relative quantification (C). Representative image of hematoxylin and eosin stained sections of aorta from both groups (D). Clustering of inflammatory cells near the intima region in the plaque of VTN siRNA treated mice (E). Scale bar = 100 µm. Aortic sections from VTN siRNA treated ApoE^−/−^ mice showing inflammatory cells (F) and recruitment of inflammatory cells from tunica media to tunica intima (G) Inset (H) shows magnified image in which ‐migration of inflammatory cells are visible. Inset (G) shows magnified image in which microvessle formation can be observed. (H) Histogram showing intima‐media thickness of aorta in both groups. (I) Graph showing the inflammation score in two groups, unpaired *t*‐test. *p < 0.05, **p < 0.01

Vitronectin gene was knocked down in vivo to see whether alteration in VTN is due to compensatory response or causative response. We questioned whether VTN downregulation could alter systemic or aortic inflammation. ApoE Knockout mice were fed with HCD for 4 weeks and then injected with si‐VTN or si‐scramble for the next 4 weeks at an interval of 10 days (Figure 4A). The downregulation of VTN expression upon si‐RNA injection was verified by western blot (Figure 4B,D). Since ApoA1 and ApoA2 are known to be involved in the inflammatory process, the plasma levels of these two proteins were determined.^27^, ^28^ Also the difference in the plasma levels of other well‐known inflammatory proteins were examined in scramble and si‐VTN. We observed elevated levels of both ApoA1 and ApoA2 as well as elevated levels of MCP‐1 and IL‐1β in VTN knockdown mice (Figure 4B). Cross‐sections of aorta stained with H & E (Figure 4D) along with its morphometric analysis (Figure 4H) revealed an increase in plaque area in VTN knockdown mice. Since VTN knockdown increased plaque deposition, the inflammatory status in the plaque microenvironment was further studied. H&E stained images of the VTN downregulated aorta showed accumulation of the inflammatory cells in the tunica media (Figure 4E,F). The migration of inflammatory cells from tunica media to tunica intima (Figure 4G) leading to microvessel formation was observed (Inset, Figure 4G). Moreover, morphometric analysis by blind investigator also revealed an increased inflammation, denoted by the inflammation score (Figure 4I) in the VTN knockdown aorta. Collectively, these data demonstrate that VTN downregulation aids in aortic deposition and inflammation.

### 3.5 VTN deficiency upregulates the expression of NF‐κB as well as adhesion molecules

**FIGURE 5:**
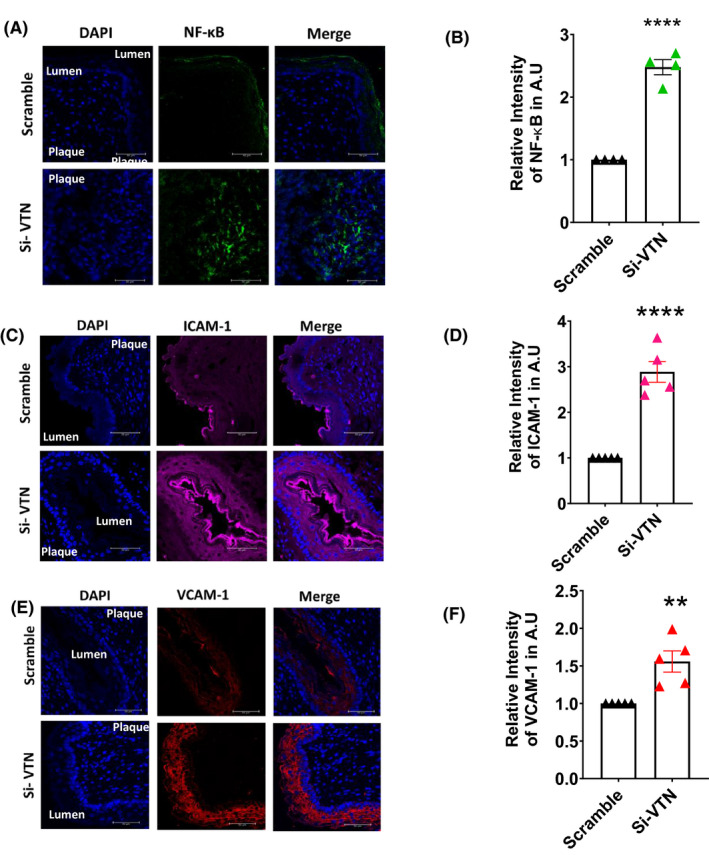
Increase in expression of NF‐κB and cell adhesion molecules upon VTN downregulation Immunofluorescence staining of mice aorta showing the increase in expression of NF‐κB (A), ICAM‐1 (C), VCAM‐1 (E), in scramble and Si‐VTN treated ApoE^−/−^ mice. Representative and the quantitative data (mean ± SEM) of relative mean fluorescence intensity from mice are presented here (B, D, F *n* = 4–6). Scale bar = 50 µm, unpaired *t*‐test. ICAM‐1, intracellular cell adhesion molecule‐1; NF‐κB, nuclear factor kappa light chain enhancer of activated B cells; VCAM‐1, vascular cell adhesion molecule‐1; VTN, vitronectin. **p < 0.01, ****p < 0.0001

To further investigate the overall inflammation in the aortic region, immunofluorescent staining of the aorta was performed. Increased inflammation in the aorta in the absence of VTN was further supported by the enhanced expression of p65‐NF‐κB (Figure 5A,B). Several reports suggest that NF‐κB induces the expression of adhesion molecules ICAM‐1 and VCAM‐1 which are detrimental to the plaque since they help in the homing of inflammatory cells and aid in atheroma formation.^29^ We found a significant upregulation of ICAM‐1 (*p* < 0.0001) (Figure 5C,D) as well as VCAM‐1 (*p* < 0.01) in VTN‐deficient mice aorta (Figure 5E,F). These data suggest that the downregulation of VTN is associated with unresolved inflammation in the plaque milieu leading to the upregulation of adhesion molecules which further aid in cellular homing at the site, leading to enhanced plaque deposition.

### 3.6 VTN deficiency alters plaque integrity

**FIGURE 6:**
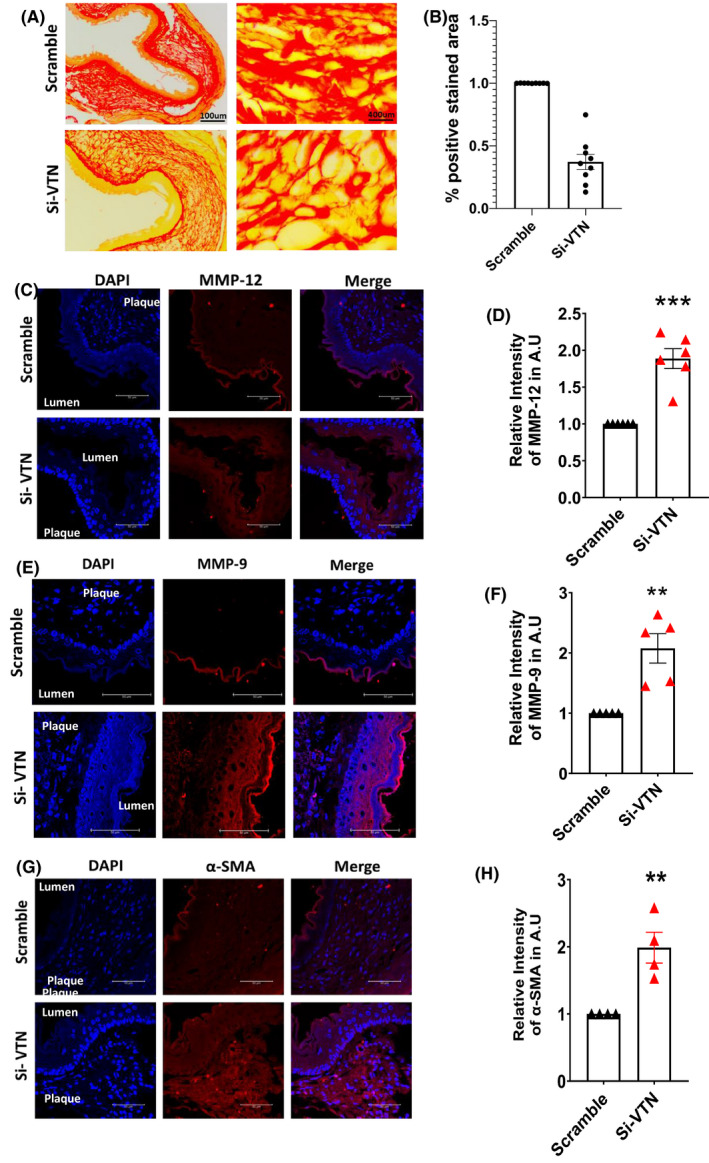
Effect of VTN downregulation in arterial remodeling (A) Picrosirius red staining of mice aorta in scramble and VTN‐siRNA treated mice (*n* = 6). (B) Graph showing the 0.36‐fold decrease in collagen content in VTN downregulated aorta. Scale bar = 100, 400 µm. Immunofluorescent staining of mice aorta showing an increase in the expression of MMP‐12 (C), MMP‐9 (E) and and α‐SMA (G) in both groups. Representative and the quantitative data (mean ± SEM) of relative mean fluorescence intensity from mice are presented here (D, F, H *n* = 4–5). Scale bar = 50 µm, unpaired *t*‐test. MMPs, matrix metalloproteases; VTN, vitronectin. *p < 0.05, **p < 0.01, ***p < 0.001

Although plaque rapture does not occur in mice, yet the integrity of the plaque was investigated by measuring collagen content by picrosirius red staining of the aorta. It was observed that collagen fibers in the plaque region were more prominent in the scramble, while in VTN knockdown mice, it was decreased to 0.36‐fold (Figure 6A,B). Matrix metalloproteinases are important determinants of plaque stability, thus the expression of both MMP‐9 and MMP‐12 in the VTN‐deficient mice aorta was examined. We observed a significant increase in MMP‐12 (*p* < 0.001) (Figure 6C,D) as well as MMP 9 expression (*p* < 0.01) (Figure 6E,F) in the VTN deficient mice aorta. α‐SMA are determinants of plaque progression and instability and they migrate into the plaque to synthesize proteins to stabilize the plaque.^30^ A significant number of lipid‐laden cells are also added by the SMCs which has been studied by colocalization studies of SMCs and lipids.^31^ VTN deficiency caused the overexpression of α‐SMA in the plaque (Figure 6G,H). Together these data suggest that the silencing of VTN is associated with the altered morphology of atherosclerotic plaque due to overexpression of α‐SMA and MMPs with reduced collagen.

### 3.7 Clinical correlation of VTN in human subjects with risk factors of atherosclerosis

**FIGURE 7:**
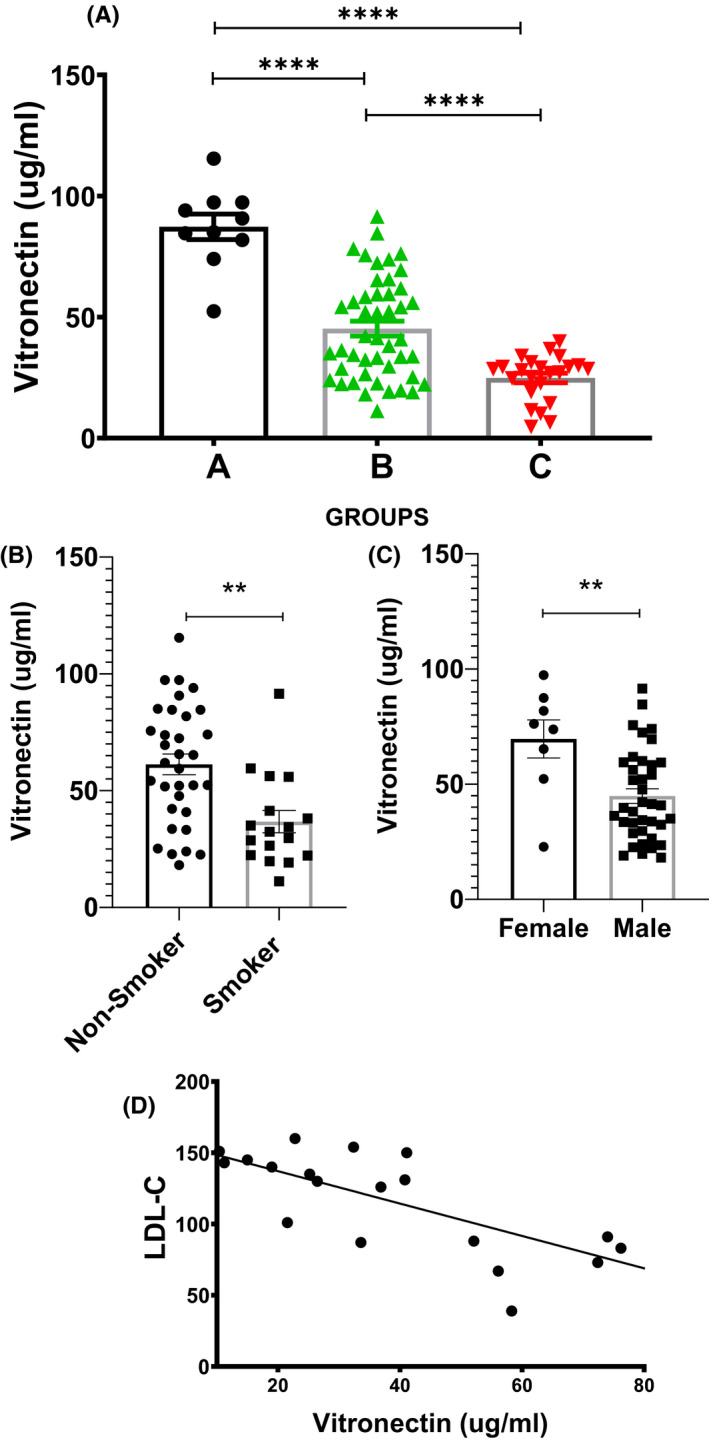
Estimation of plasma level of vitronectin (VTN) in human subjects and its correlation with risk factors associated to coronary artery disease (CAD). (A) Illustrative dot plot showing levels of VTN in plasma of Group A (Healthy control, *n* = 10), Group B (Control with risk factors, *n* = 46) and Group C (acute coronary syndrome patients, *n* = 23). (B) Scatter Plot showing the plasma levels of VTN in non‐smoker (*n* = 30) versus smokers (*n* = 16). (C) Scatter Plot showing the plasma levels of VTN in female (*n* = 8) and male (*n* = 38) subjects. (D) Scatter plot showing the correlation between VTN levels and low‐density lipoprotein‐cholesterol (LDL‐C) in human aged above 40 years (Pearson correlation coefficient = −0.74, *n* = 19), unpaired *t*‐test. **p < 0.01, ****p < 0.001

Since VTN is downregulated due to high cholesterol load, we were interested to verify the same in human subjects. The baseline characteristics of the study population are provided in Table 1. As shown in Figure 7A, the circulatory levels of VTN were significantly (*p* < 0.0001) decreased in individuals with risk factors of atherosclerosis (45.23 ± 3.08 µg/ml) compared to healthy control (87.26 ± 5.25 µg/ml). Moreover, there was a severe reduction of VTN level in individuals having ACS (24.85 ± 1.98 µg/ml) compared to healthy control (*p* < 0.0001). This data indicates VTN level is inversely related to the severity of CAD. Since VTN levels were decreased in subjects with ACS, any relationship with the risk factors of atherosclerosis was also examined. There was a significant reduction of VTN levels (*p* < 0.01) in smokers (36.70 ± 4.77 µg/ml) compared to nonsmokers (61.24 ± 4.43 µg/ml) (Figure 7B) and also in males (44.82 ± 3.1 µg/ml) compared to females (69.61 ± 8.24 µg/ml) (Figure 7C). It was found that VTN level was inversely correlated with the plasma level of LDL cholesterol in subjects above 40 years (*r* = −0.74), *p* = 0.0002 (Figure 7D). Taken together the results depict an inverse relationship between plasma VTN levels and atherosclerotic risk factors.

**TABLE 1:** Baseline characteristics of study population

Variables	Group A (*n* = 10)	Group B (*n* = 46)	Group C (*n* = 23)
Age	41.8 ± 9.99	46.45 ± 11.83	57.13 ± 12.27
Total cholesterol (mg/dl)	170.7 ± 14.94	194.71 ± 46.51	207.44 ± 41.06
LDL cholesterol (mg/dl)	100.3 ± 13.02	113.90 ± 33.85	123.55 ± 21.22
HDL cholesterol (mg/dl)	48.2 ± 7.00	45.11 ± 9.91	41.44 ± 6.48
Triglycerides (mg/dl)	108.7 ± 20.33	182.47 ± 124.72	180 ± 61.28

## 4 DISCUSSION

In this study, the effect of VTN deficiency in athero‐progression and athero‐inflammation was assessed. We demonstrate that chronic cholesterol load suppresses the expression of VTN in the liver. This response causes acceleration of atherosclerosis, mediated by the enhanced inflammation and induction of adhesion molecules. Increased homing of inflammatory cells and altered morphology, challenges the structural stability of the plaque by overexpressing MMPs. Our study establishes that VTN regulates cholesterol‐driven atherosclerosis and inflammation in the local milieu of the arteries, establishing a pivotal role of VTN in the pathogenesis of CAD.

Inflammation and atherosclerosis have a complex relationship where inflammation plays a vital role in subsequent plaque development.^1^ Such inflammatory process in the progression and development of plaque is primarily mediated by the interaction of different immune cells with damaged endothelium, which is controlled by various types of cell surface proteins including extracellular matrix proteins and CAMs.^10^, ^32^ Additionally, extracellular matrix proteins also play an important role in the pathophysiology of atherosclerosis and behave like a biological glue that holds cells together, which might be critical for plaque progression and myocardial complications.^33^, ^34^ Thus, understanding the role of adhesion molecules and the cellular as well as molecular events associated with developmental atherosclerosis is important for the strategic development and management of CAD.

Earlier studies showed that VTN is present in the aortic wall of the rabbit aorta indicating its importance in atherosclerosis.^35^ In the present study, the plasma level of VTN is decreased after 45 days with an initial increase after 30 days in ApoE knockout mice. Such initial rise of VTN expression appears to be a compensatory response suggesting it could be beneficial for maintaining the normal physiological conditions in the arterial plaque. So the initial increase of VTN in our study, and increased VTN that was observed in other cross‐sectional studies could be a compensatory mechanism to protect the cells.^23^, ^24^ However, this protective mechanism may fail in chronic cholesterol load since a mechanistic analysis suggested that the downregulation of VTN, is caused partly due to the regulatory effects of cholesterol on its promoter region. The exact molecular mechanism for cholesterol‐mediated VTN gene expression is not known and whether SREBPF or any other proteins are involved in such regulation needs more investigation in the future. The causal role of cholesterol in the downregulation of VTN is further verified in PPARα Knockout mice in which the endogenous level of cholesterol remains elevated due to impaired cholesterol metabolism in addition to triglyceride.^36^, ^37^ Cholesterol and LDL‐C mediated downregulation of VTN is further validated in vitro. The reciprocal relationship between cholesterol and VTN was confirmed both in vitro and in vivo, where atorvastatin significantly induces the expression of VTN. Since chronic CAD patients are regularly treated with statins for the management of blood cholesterol levels, VTN levels might be higher in CAD subjects as reported in earlier studies. Taken together it is likely that due to possible management of cholesterol levels upon statin administration, VTN level is maintained, which may be one of the mediators of the beneficial effects of the cholesterol‐lowering drugs on the progression of atherosclerosis.

The time kinetics experiments revealed the reduction of VTN after 45 days of high cholesterol feeding indicating that reduction seems to be the effect of chronic hypercholesterolemia. Since there was a transient increase in VTN in response to HCD at earlier time points, we hypothesize this to be a compensatory response that might have been lost upon continuous feeding of cholesterol. To establish the causal effect of VTN in atherosclerotic plaque, si‐RNA mediated silencing of the VTN gene was performed in ApoE knockout mice. The results demonstrate that VTN knockdown accelerates atherosclerotic progression. In the aorta, the infiltration of inflammatory cells occurs along with the microvessel formation in VTN‐deficient conditions. Earlier reports demonstrate the activation of NF‐KB in VTN deficient neutrophils.^38^ Immunofluorescence studies also revealed the overexpression of NF‐KB at the site of the plaque, responsible for driving the plaque towards a chronic inflammatory state. Both ApoA‐I and ApoA‐II are upregulated in plasma upon VTN silencing. Apol‐I reduces inflammation by inhibiting the transendothelial migration of inflammatory cells and by reducing the expression of integrins, while ApoA‐II has been found to induce acute phase response in mice leading to accelerated atherosclerosis.^28^, ^39^ Increased ApoA‐I levels might be a response to combat the inflammation, on the other hand, an increase in ApoA‐II further supports accelerated atherogenesis. We also found elevated levels of interleukin‐1β (IL‐1β) as well as monocyte chemotactic protein‐1 (MCP‐1) in VTN deficient mice. MCP‐1 is regarded as a major chemotactic molecule which is generated within the vessel and is responsible for the recruitment of different monocyte, neutrophil and lymphocytes to the site of the plaque.^40^ Additionally, reports suggest that IL‐1β is responsible for inducing atherosclerosis.^41^ These findings further support that VTN reduction drives the system to more atheroprone. Transendothelial migration of the different immune cells critically regulates the initiation, deposition, inflammation, and plaque rupture. These events signify the role of ICAM‐1 and VCAM‐1 in atherosclerosis, which is induced by NF‐KB.^42^, ^43^, ^44^, ^45^, ^46^, ^47^, ^48^ Our data demonstrate that VTN deficiency induces the migration of inflammatory cells by upregulating ICAM‐1 and VCAM‐1 expression.

Guided by these observations, the collagen deposition in the plaque was monitored since it is known to help in the stabilization process^49^ The stability of the plaque is unequivocally important in determining acute events which result from plaque rupture. Though plaque rupture in the real sense does not occur in mice, yet the depleted collagen content in the plaque area in VTN deficient mice may pose a challenge to the morphological and structural integrity. Matrix metalloproteases are the main enzymes that disrupt the triple helix and cause collagen lysis.^50^ Our studies revealed a marked increase in MMP‐9 and MMP‐12, which are prominent predictors of plaque instability.^51^, ^52^, ^53^ This data further justifies the low level of VTN in subjects with ACS compared to healthy controls. We also observed a significant decrease in VTN levels in subjects having risk factors for atherosclerosis. The plasma level of VTN is significantly lower in smokers compared to non‐smokers. It is established that males are more prone to atherosclerosis compared to females.^54^, ^55^ Consistent with this, higher VTN levels were observed in human females.

It is reported that free cholesterol particles which are released from atheromatous plaque, act as complement activators that generate MAC leading to plaque destabilization. Though MAC is traditionally believed to have a role in microbial infections, complement‐induced MAC is crucial in the progression of atherosclerosis in ApoE knockout mice. VTN inhibits MAC formation.^26^, ^56^ So reduced VTN in ACS subjects could be attributable to less inhibition of complement‐mediated formation of MAC.^57^ As a result, a complement‐mediated injury to structural cells of the aorta could occur, which might cause injury and further challenge the integrity of atherosclerotic plaque. So, the initial compensatory increase of VTN might be to inhibit the MAC and thus protect the cells. It is important to observe that medications might influence the levels of VTN. Apart from this exercise, lifestyle, food habits, cholesterol load, and other cardiovascular risk factors such as insulin resistance, hyperinsulinemia, etc. might have a role in the expression of this protein, which warrants further study.

**FIGURE 8:**
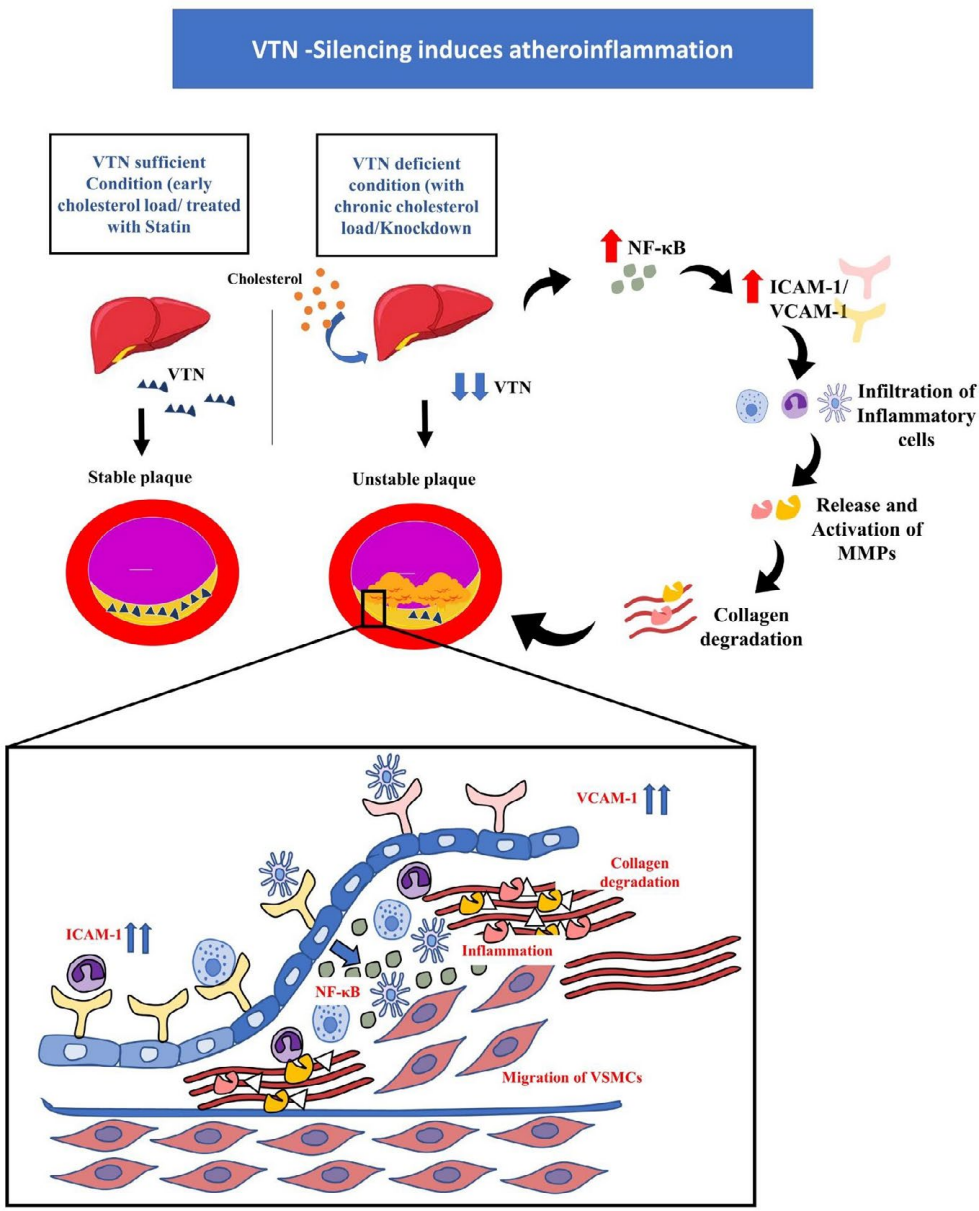
Schematic representation depicting the role of VTN in atherosclerotic plaque. The supply of sufficient VTN in the atherosclerotic plaque maintains stable conditions in the plaque, while chronic cholesterol load causing VTN downregulation induces a series of events which initiates inflammation leading to upregulation of NF‐κB, which in turn enhances ICAM‐1 and VCAM‐1 secretion. This leads to inflammatory cells build up and a shifting towards unresolved and chronic inflammation in the plaque mileu. In addition, MMPs are activated which leads to severe collagen degradation, resulting in an unstable plaque, indicating vulnerability towards acute events. Up arrow indicates upregulation and down arrow indicates down regulation. ICAM‐1, intracellular cell adhesion molecule‐1; MMPs, matrix metalloproteases; NF‐κB, nuclear factor kappa light chain enhancer of activated B cells; VCAM‐1, vascular cell adhesion molecule‐1; VSMC, vascular smooth muscle cells

The current literature has indicated VTN as an adhesion molecule only. Our findings have unveiled a multi‐functional role of VTN beyond adhesion function. Although, VTN was reported to be localized in the atherosclerotic wall of cardiac patients in rabbits and its role in CAD was emphasized in the context of platelet abnormalities including platelet activation & aggregation, however, systematic study and dynamic relationship of VTN with the progress of atherosclerosis were not known.^14^, ^35^As there is limited scope for interventional study in humans, complementary mice studies with the atherosclerotic model are the best option. This study tried to fill the gaps in the existing knowledge with time kinetic as well as interventional studies. Dysregulated cholesterol has two distinct but overlapping implications in the cardiovascular system that include atherogenesis and inflammation that eventually triggers thrombotic plaque complications. Our data indicate, VTN might have some protective role in CAD. Imbalance in the levels of VTN facilitates the trafficking of the inflammatory cells to the plaque microenvironment. When VTN is repressed due to high cholesterol load, aortic inflammation is driven towards an unresolved state leading to the advanced necrotic atheroma that might cause myocardial infarction (Figure 8). However, a large‐scale and longitudinal cohort study may be required to clinically correlate plasma VTN level with atherosclerotic burden and acute coronary events in human subjects.

## CONFLICT OF INTEREST

The authors declare no conflict of interest.

## AUTHOR CONTRIBUTIONS

Devasmita Chakravarty conducted experiments, analyzed data, drafted the manuscript. Aleepta Guha Ray, Vivek Chander, Aditya Konar, and Bishnu Prasad Sinha conducted experiments. Ulaganathan Mabalirajan conducted experiments and edited the manuscript. Prakash C. Mondal designed clinical studies, collected human patient samples, analyzed data, and edited the manuscript. Khawer N. Siddiqui Designed clinical studies, collected samples from healthy control, analyzed data and edited the manuscript. Arun Bandyopadhyay conceptualized, analyzed data, managed resources, and edited manuscript.

## ETHICAL STATEMENT

All human and animal studies have been approved by the appropriate ethics committee and have therefore been performed following the ethical standards laid down in the 1964 Declaration of Helsinki and its later amendments.
